# Altered responses to ghrelin and food cues in AgRP neurons during pregnancy and lactation

**DOI:** 10.1111/jne.70202

**Published:** 2026-06-09

**Authors:** C. L. Murrell, M. R. Perkinson, Z. B. Andrews, D. R. Grattan, S. R. Ladyman

**Affiliations:** ^1^ Department of Psychiatry Washington University in St. Louis St Louis Missouri USA; ^2^ Centre for Neuroendocrinology and Department of Physiology School of Biomedical Sciences, University of Otago Dunedin New Zealand; ^3^ Monash Biomedicine Discovery Institute and Department of Physiology Monash University Clayton Australia; ^4^ Centre for Neuroendocrinology and Department of Anatomy, School of Biomedical Sciences University of Otago Dunedin New Zealand; ^5^ Maurice Wilkins Centre for Molecular Biodiscovery Auckland New Zealand

**Keywords:** AgRP neurons, arcuate nucleus, ghrelin, pregnancy

## Abstract

Pregnancy and lactation trigger many metabolic adaptations, including increased food intake to support the energy demands of the growing foetus and then to provide nutrition through milk production after birth. Ghrelin, an orexigenic hormone, activates agouti related peptide (AgRP) neurons in the arcuate nucleus to promote food intake. Here, we investigated the hypothesis that increased sensitivity to ghrelin during pregnancy and lactation may contribute to elevated maternal food intake. Acute food intake was measured after a single dose of ghrelin or vehicle across reproductive states including virgin, pregnant (Day 8 and Day 15), lactating (Day 10) mice and dams 2 weeks after weaning. In vivo GCaMP fibre photometry of the AgRP neuron population was used to measure AgRP neuronal response to ghrelin. Unlike virgin mice, pregnant mice did not show an acute increase in food intake after ghrelin injection, while ghrelin‐treated lactating mice showed a greater feeding response than virgin mice. After weaning, dams showed a similar increase in food intake to that seen in virgin mice. In contrast to the loss of feeding response to ghrelin, the expected increase in growth hormone (GH) in response to ghrelin was observed in both pregnancy and lactation. Across all of the reproductive states, a significant increase in AgRP neuron activity was observed in response to exogenous ghrelin administration, although the magnitude was slightly reduced in late pregnancy. Furthermore, the ghrelin‐induced increase in c‐Fos expression in AgRP neurons was similar in all reproductive states, indicating that AgRP neurons remained responsive to ghrelin despite the absence of a food‐intake response to ghrelin during pregnancy. Interestingly, the expected drop in AgRP neuron activity in response to the presentation of food was absent during late pregnancy and lactation. The absence of a food consumption‐mediated inhibition of AgRP neuron activity suggests that an attenuated response of the AgRP neurons to feedback signals associated with eating may contribute to increases in meal duration during pregnancy and lactation. Overall, these results indicate that ghrelin resistance develops during pregnancy, suggesting that ghrelin does not contribute to elevated food intake during pregnancy. In lactation, however, enhanced ghrelin sensitivity may contribute to elevated maternal food intake. These results also indicate that adaptations to ghrelin sensitivity in pregnancy and lactation are transient as 2 weeks after weaning our results are similar to the virgin state.

## 1 INTRODUCTION

Pregnancy and lactation are energetically demanding physiological states. During pregnancy, the mother must supply all the energy required for foetal growth as well as to support changes in her own physiology. In lactation, the mother continues to meet the nutritional demands of offspring via milk production. In most mammals, food intake increases during pregnancy and lactation to support these new energy demands.

Arguably, the increase in food intake during lactation is driven in response to the metabolic load of milk production. In pregnancy, however, increased food intake is an adaptive, anabolic state, contributing not only to the maintenance of pregnancy but also to fat deposition in anticipation of the future metabolic demands of lactation. Supporting this adaptive state, many anorectic hormones fail to suppress food intake during pregnancy (reviewed in^1^), facilitating elevated food intake. For example, levels of the anorectic hormone leptin increase during pregnancy, due to placental production, increased fat mass and/or lower clearance from the blood.^2^, ^3^, ^4^, ^5^, ^6^ Despite this elevation in circulating leptin, food intake increases due to an insensitivity to the satiety actions of leptin that develop by mid pregnancy.^1^, ^7^, ^8^

The orexigenic hormone ghrelin is produced and secreted by the stomach, and it is the only peripherally‐derived hormone that stimulates food intake.^9^, ^10^ Ghrelin concentrations in the blood increase prior to meals^11^ and contribute to promoting hunger and food consumption.^12^, ^13^ Acute administration of ghrelin in rodents and humans, either by intracerebroventricular (i.c.v.) or intraperitoneal (i.p.) injection, results in a rapid increase in food intake compared to vehicle treatment. Studies measuring total ghrelin levels in humans, mice and rats report decreased ghrelin in pregnancy.^14^, ^15^, ^16^, ^17^ The few studies measuring acyl‐ghrelin, the active form, report either increases (rats and humans)^18^, ^19^ or decreases (mice and humans).^15^, ^20^ Here, we measure the downstream responses to ghrelin administration.

Within the brain, the agouti‐related peptide (AgRP)/neuropeptide Y (NPY) neurons (AgRP neurons henceforth) found in the arcuate nucleus of the hypothalamus^21^ are a key neuronal population driving food intake and motivated food seeking.^22^, ^23^, ^24^ This population acts as the neural substrate of hunger^23^, ^25^ and optogenetic or chemogenetic stimulation of these neurons directly increases food intake,^26^, ^27^ whereas ablation of these neurons in adults reduces food intake.^28^, ^29^ Both neuropeptides, AgRP and NPY, are orexigenic, and this neuronal population also stimulates food intake via release of GABA.^23^ Temporal regulation of food intake is differentially modulated by these neuropeptides and neurotransmitters. Rapid stimulation of food intake is mediated by NPY and GABA, whilst slower sustained increases in food intake are driven by AgRP via the melanocortin pathway.^30^, ^31^, ^32^, ^33^ AgRP neurons integrate a wide range of signals, including ghrelin, to generate an appropriate hunger drive.^23^ The ghrelin receptor, growth hormone‐secretagogue receptor (GHSR), is expressed in over 90% of the AgRP neurons^34^ and is vital for ghrelin‐induced food intake and AgRP neuron stimulation.^35^, ^36^ In this study, we have characterised hypothalamic and behavioural responses to ghrelin in pregnancy and lactation, to test the hypothesis that an increase in sensitivity to ghrelin might contribute to elevated food intake in pregnancy and lactation. Alongside this, we investigated whether pregnancy and lactation induce adaptations in acute responses of AgRP neurons to food‐related cues. Using in vivo calcium‐dependent fibre photometry, the acute responses of the AgRP neuronal population to various stimuli (ghrelin, standard rodent chow and highly palatable food)^24^, ^37^, ^38^, ^39^, ^40^, ^41^, ^42^ were assessed in the non‐pregnant state, during pregnancy and lactation, and after dams had weaned their pups.

## 2 METHODS

### 2.1 Animals

All experiments were approved by the University of Otago Animal Ethics Committee and conducted in accordance with the New Zealand Animal Welfare Act (1999) and associated guidelines. Mice were at least 8 weeks of age before any experiments began and were group housed until start of experimental protocols. Mice were housed at room temperature of 21 ± 1°C, with a 12 h light:12 h‐dark light cycle (07:00–19:00 h) and had ad libitum access to standard rodent chow (#2918 Teklad Irradiated Global 18% Protein Rodent Diet; 18% protein, 6% fat, 44% carbohydrate) and water, in either open top cages or individually ventilated cages. Food intake and growth hormone (GH) tests were completed on wild‐type C57BL/6J adult female mice, obtained from the University of Otago's breeding colony at the Biomedical Resource Unit. For in vivo fibre photometry studies, *Agrp*‐ires‐cre mice were obtained from Jackson laboratory (stock no. 012899).^43^ For c‐Fos detection in AgRP neurons, *Agrp*‐ires‐cre mice were bred with Cre‐dependent td‐Tomato reporter mice obtained from Jackson laboratories (stock no. 007909),^44^ resulting in *Agrp*‐ires‐cre × td‐Tomato mice. For all experiments, timed pregnancies were generated by housing each female mouse with a C57BL/6J stud male. The presence of a vaginal plug was deemed Day 1 of pregnancy, and the day of parturition (generally Day 19 or 20 of pregnancy in our colonies) was deemed Day 1 of lactation. On Day 3 of lactation all litters were normalised to 6 pups per dam. Pups were weaned on day 20 of lactation by removal from the dam's cage. When required for virgin experimental days, daily vaginal cytology was used to determine the stage of the oestrous cycle.^45^

### 2.2 Ghrelin‐induced food intake, behaviour and GH secretion

Body weight and food intake of adult female C57BL/6J mice were measured daily between 08:00 and 09:00 h for each mouse, before (7 days prior to mating), during (Days 1 to 18 of pregnancy) and after pregnancy (from Day 3 of lactation to 2 weeks after weaning of the dam's pups). At specific reproductive timepoints (virgin metoestrus, Days 8 and 15 of pregnancy, Day 10 of lactation and 2 weeks after weaning) mice underwent a ghrelin‐induced feeding response paradigm. Food was removed from the home cage at 09:00 h then 5 h later mice received an i.p. injection of ghrelin (0.3 mg/kg dissolved in saline 10 μL/g, B0084‐103854 rat ghrelin, Best of Chemicals sciences, NY, USA)^24^, ^42^ or vehicle (saline, 10 μL/g). Three pellets of food (~ 15 g) were returned at the time of injection and amount of food consumed over the following 2 h was measured by weighing the pellets left in the cage. Mice were randomly selected to receive either vehicle or ghrelin at each timepoint. Approximately half of the mice in this study were not treated on Day 8 of pregnancy due to the timing of the New Zealand Covid 19 lockdown. Importantly, in the lactating groups, pups were not removed for this feeding‐response paradigm, which complicated interpretation (discussed further in the results section, below).

In a separate cohort of lactating dams together with their pups (*n* = 20, plus 6 pups each), home cage activity for the 2 h after injection of ghrelin or vehicle was recorded and analysed for time spent engaged in various behaviours including pup interaction, food interaction, and being off the nest which includes digging, self‐grooming, rearing and drinking. For analysis, behaviour of each experimental mouse was determined every 60 s for the duration of the 2 h period to give a total of 120 data points.

To assess the GH response to ghrelin, another cohort of mice (female C57BL/6J, *n* = 18) were habituated for 2 weeks to daily handling for serial tail tip blood sampling.^46^ Oestrous cycles were monitored daily by vaginal cytology and on metoestrus or dioestrus, food was removed at 09:00 h. Approximately 3 h later, 4 μL of whole blood was collected from the tail tip every 10 min for 50 min (six samples in total). After the second blood sample was collected, mice received an i.p. injection of either ghrelin or vehicle as described above. After the final blood sample collection, food was returned to the cage and approximately 5 days later, mice were housed with a stud male to generated timed pregnancies. On Day 15 of pregnancy, mice underwent 3 h of food removal from cage, followed by collection of six blood samples every 10 min, with an injection of either ghrelin or vehicle after the collection of the second blood sample, as described above. During the blood‐sampling period, whole blood samples were immediately placed in 96 μL of 0.01 M phosphate buffered saline with 0.05% Tween‐20, frozen on dry ice and stored at −70°C until further processed for determination of GH concentration using an ultra‐sensitive enzyme‐linked immunosorbent assay (ELISA). This was an in house ELISA, previously validated and described,^46^ based on reagents from the National Institute of Diabetes and Digestive and Kidney Diseases (NIDDK)‐National Hormone and Pituitary Program (NHPP, Torrance, CA). The capture antibody was monkey anti‐rat GH (anti‐rat GH‐S‐5, AFP411S; 1:40,000 dilution), and the detection antibody was rabbit anti rat GH (AFP5672099; 1:40,000), followed by incubation with horseradish peroxidase‐conjugated antibody (Polyclonal goat anti‐rabbit immunoglobulin, P0448, DakoCytomation) at a final dilution of 1:2000. The standard curve was established using mouse GH reference preparation (AFP‐10783B). The intra‐ and intraassay coefficients of variation were < 10% and < 15%, respectively. Area under the curve (AUC) was used to determine GH release in response to ghrelin. The GH concentration in response to ghrelin was normalised to the GH hormone concentration in response to vehicle at the same reproductive state.

### 2.3 Viral delivery and fibre optic placement for assessment of acute AgRP neuronal activity in response to ghrelin and food

Adult *Agrp*‐ires‐cre female mice (8–10 weeks old) were given prophylactic carprofen for pain relief (5 mg/kg, s.c.), then anaesthetised with 2%–3% isoflurane and placed in a stereotaxic frame. A unilateral injection of Adeno‐associated virus (AAV) encoding GCaMP6s (AAV9.CAG.Flex.GCaMP6s.WPRE.SV40 1 × 10^13^ vg/ml, Addgene, University of Pennsylvania Vector Core) was administered into the arcuate nucleus (−0.9 mm AP, +0.4 mm ML, −5.8 mm DV relative to Bregma) via a Hamilton syringe at a volume of 1 μL over 10 min. A fibre optic cannula (400 μm, NA 0.48, MFC_400/430‐0.48_6.5 mm_SM3*_FLT, Doric Lenses, Canada) was then implanted 0.1 mm above the AAV injection site and secured to the skull using adhesive dental cement. Mice were left to recover for 5 days with additional doses of carprofen (5 mg/kg, s.c.) given daily for the first 3 days. For the next 2 weeks mice were handled daily, followed by 1 week of habituation to the tethering of the fibre optic cannula to the fibre photometry set up, including exposure to the presentation of a peanut butter chip (PB) (Reece's PBs; fat 29% carbohydrate 52% protein 3%, Hershey, Pennsylvania, USA) (~15 mg) and a white plastic block (5 mm^3^), before experimental recordings took place.

### 2.4 Fibre photometry system

Changes in fluorescence from the population of AgRP neurons were detected using custom acquisition software with hardware components purchased from Doric Lenses.^47^ Excitation LEDs at 405 nm (violet) and 465 nm (blue) were sinusoidally modulated at 531 and 211 Hz, respectively. Each excitation wavelength passed through a mini cube excitation filter (405 nm and 460–490 nm) and focused into a 400 μm (NA 0.57) optic fibre connected to the fibre optic implant (NA 0.48) in the mouse. Light power for the 465 nm output at the tip of the fibre was set to 80–100 μW. The 405 nm wavelength reported calcium‐independent signalling (background) at the GCaMP6s isosbestic point, and 465 nm signal reported calcium‐dependent GCaMP6 excitation to infer changes in intracellular calcium. A single emission from GCaMP6s in AgRP neurons was collected by the same fibre optic probe and passed through a 500–550 nm emission filter (Fluorescence Mini cube, Doric Lenses) then focused onto a photoreceiver (Newport 2151, Doric Lenses). The signals were demodulated and recorded at 10 Hz with custom acquisition software (Tussock innovation, New Zealand).

### 2.5 Experimental paradigms to assess AgRP neuron population activity

**FIGURE 1:**
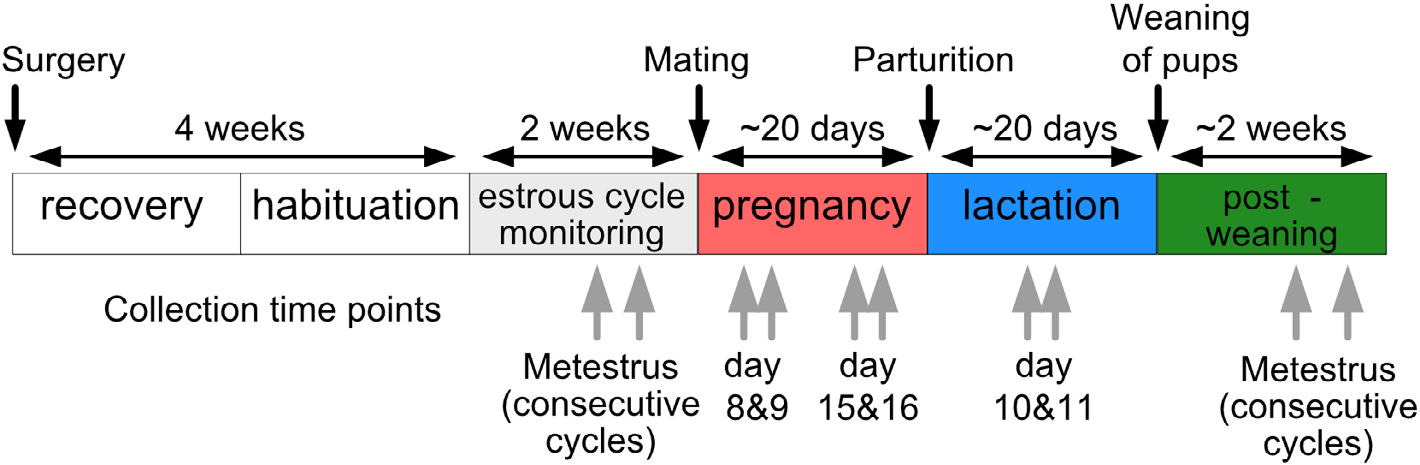
Experimental timeline. Outline of protocol for examining agouti related peptide (AgRP) neuronal population activity using GCaMP6 fibre photometry across pregnancy and lactation. Grey arrows indicate timepoints of fibre photometry recordings.

All experiments were conducted in the home cage with food removed. All tests were video recorded with a webcam (Logitech 4 k Pro) to enable alignment of behaviour with changes in AgRP neuronal activity. Recordings were carried out between 07:00 and 12:00 h. As illustrated in Figure 1, the same mice were used across all reproductive states. These mice underwent the test‐day paradigms on metoestrus of two consecutive oestrous cycles, on Days 8/9 of pregnancy, Days 15/16 of pregnancy, Days 10/11 of lactation and metoestrus 2 weeks post weaning. The test was a 2‐day protocol recording AgRP neuron activity in response to four different parameters with a cross‐over design. On Day 1, mice were exposed to either PB or white plastic block (control), followed by either ghrelin or vehicle administration then presentation of chow 30 min later. On Day 2, mice were exposed to the item they had not been presented on Day 1 (PB or white plastic block) and then received the treatment (ghrelin or vehicle) that they had not received the day before. The order of treatment and control parameters was pseudorandomised across each reproductive state. To avoid the confound caused by the presence of pups, lactating animals had their pups removed from the home cage for the duration of the photometry recordings, and the pups were returned to the dams at the completion of recordings.

On the test day, mice were connected to the fibre photometry set up via a patch cord and given a 15 min acclimatisation period before a 10 min baseline recording was taken. Then, either PB (15 mg) or a white plastic block was dropped into the home cage and the response was recorded for 10 min. Following this, a new 10 min baseline was recorded before injection (i.p.) of either vehicle or ghrelin (0.3 mg/kg). After 10 min, standard laboratory animal chow was given to the mice, and their neuronal response was recorded for a further 30 min. A second recording with the alternate parameters was completed on metoestrus of the subsequent oestrous cycle for non‐pregnant animals or on the following day in pregnancy and lactation.

For each recording, calcium‐independent signal (405 nm) was subtracted from the calcium‐dependent signal (490 nm) before analysis. To correct for bleaching, the slope of 405 nm signal was fitted against the 465 nm signal where $∆F/F=\frac{465\;\mathrm{nm}-\text{fitted}\;405\;\mathrm{nm}}{\text{fitted}\;405\;\mathrm{nm}}$. Individual *z*‐scores were used to normalise the baseline period of activity before each manipulation to allow comparison of each event across multiple mice. To calculate *z*‐score the mean ($\mu_{\text{baseline}}$) and standard deviation $\left(\sigma_{\text{baseline}}\right)$ of ∆F/F were computed from a stable 9 min and 30 s, where the manipulation occurs at 10 min (30 s was removed from baseline due to movement and handling of the mouse to give the i.p. injection). Subsequent data were *z*‐scored using the formula $z=\frac{∆F/F-\mu_{\text{baseline}}}{\sigma_{\text{baseline}}}$. Mice were only included in the study if, after normalisation, ghrelin treatment in the nonpregnant state achieve a *z*‐score of 4 or more.^24^ To compare responses between reproductive states, *z*‐scores were averaged over the 10 min response time. By using *z*‐scores, as oppose to raw photometry recordings, we were able to normalise for potential changes in signal quality both between animals, and within animals over time throughout the longitudinal design of this study. Time zero was defined as the time of injection of ghrelin or vehicle, or as time of first contact with PB or chow for feeding responses. Anticipatory activity was analysed as the 60 s preceding chow contact and the 30 s preceding PB contact. These anticipatory periods were based on the rounded average latency from the introduction of food into the cage to the initial contact of the mouse with the food. A custom‐built Python application was used to align all fibre photometry data with coded events, calculate the AUC in 5 s bins using the simpson function from scipy.integrate Perkinson, M. (2024). NeuroSyncApp, v2.0.15 [Software]. Zenodo. https://doi.org/10.5281/zenodo.14254094. Updated versions available on GitHub: https://github.com/Michael-Perkinson/NeuroSyncApp.

### 2.6 Viral transfection and optic fibre placement assessment

Mice were anaesthetised with a pentobarbital overdose and once the pedal withdrawal reflex was lost, they were perfused with 4% paraformaldehyde (PFA) in a phosphate buffer (0.1 M, pH 7.6); the brains were immediately removed and placed in 4% PFA overnight. The following day, brains were transferred to 30% sucrose solution in phosphate buffer (0.1 M, pH 7.6) and stored at 4°C until they were infiltrated with sucrose. Coronal brain sections (30 μm) were cut through the brain using a sliding microtome at −17°C and three series of sections were collected through the arcuate nucleus (bregma −0.9 mm – bregma −2.5 mm coronal sections). One series of free‐floating sections from each mouse underwent immunohistochemistry for detection of green fluorescent protein (GFP),^48^ indicative of viral transduction of GCaMP6s.^39^ Briefly, after incubation to quench endogenous peroxidase (1 × TBS, methanol and 30% H_2_O_2_), sections were incubated in rabbit anti‐GFP antibody (Catalogue no. a6455 1:5000, Life Technologies, CA, USA) diluted in 0.3% Triton X‐100, 0.25% bovine serum albumin, and 2% normal goat in TBS for 48 h at 4°C followed by a 3 h incubation in goat anti‐rabbit 488 immunoglobulin (1:500, Invitrogen, MA, USA). Sections were mounted on gelatine coated slides and covered slipped with Fluoromount G (Southern Biotech, Birmingham, Alabama). The slides were viewed using the Olympus BX51 epifluorescent microscope (Olympus, Tokyo, Japan).

### 2.7 Ghrelin‐induced c‐Fos in AgRP neurons

Additional groups of virgin, Day‐15 pregnant and Day‐10 lactating *Agrp*‐ires‐cre × td‐Tomato mice were injected (i.p.) with either ghrelin (0.3 mg/kg) or vehicle (saline) in the early light phase of the light cycle (0700 h – 1000 h). Food was removed at the time of injection and after 2 h their brains were collected and processed as described above.

One series of free‐floating sections from each brain was processed for c‐Fos immunofluorescence using tyramide amplification as previously described.^49^ Briefly, sections were incubated in rabbit anti‐c‐Fos antibody (Catalogue no. ab190289, RRID:AB_2737414 1:5000, abcam, Cambridge, UK) for 48 h at 4°C followed by 1 h in biotinylated goat anti‐rabbit immunoglobulin (Catalogue no. BA‐1000, RRID:AB_2313606 1:200, Vector Laboratories, CA, USA), then incubated with biotinylated tyramide (Catalogue no. B40951 Invitrogen, MA, USA) for 20 min at room temperature followed by a 2 h incubation at 37°C in streptavidin 488 fluorophore (Catalogue no. s11223 1:400, Thermo Fisher Scientific, MA, USA). Sections were mounted on gelatine coated slides and covered slipped with Fluoromount G (Southern Biotech, Birmingham, Alabama). The slides were viewed using the Olympus BX51 epifluorescence microscope (Olympus, Tokyo, Japan) with 10 x and 20 x objectives and images were obtained using PROGRES GRYPHAX digital camera and software (Jenoptik, Jena, Germany). Analysis of images was completed using *FIJI* version 2.9.0 (Image J) software. Using the *FIJI*'s pencil tool a border was drawn around the region of interest and using *FIJI's* multipoint tool each immuno‐reactive cell was counted. During this process the analyser was blinded to experimental group.

### 2.8 Statistical analysis

Data are represented as mean ± standard error of the mean (SEM). All statistical analyses were performed using GraphPad Prism version 10.5.0. and *p* < 0.05 was considered statistically significant. All statistical outcomes are reported in the statistics table (Table S1). In general, data were analysed one‐way repeated‐measures ANOVA where 3 groups were present. When comparing conditions (i.e., Ghrelin vs. vehicle) across groups, a two‐way repeated‐measures ANOVA or Mixed Model ANOVA were used. Comparison of GH AUC was done by paired *t*‐test. Post hoc analysis was completed where appropriate and is outlined in Table S1.

## 3 RESULTS

### 3.1 Effect of ghrelin in the virgin state

**FIGURE 2:**
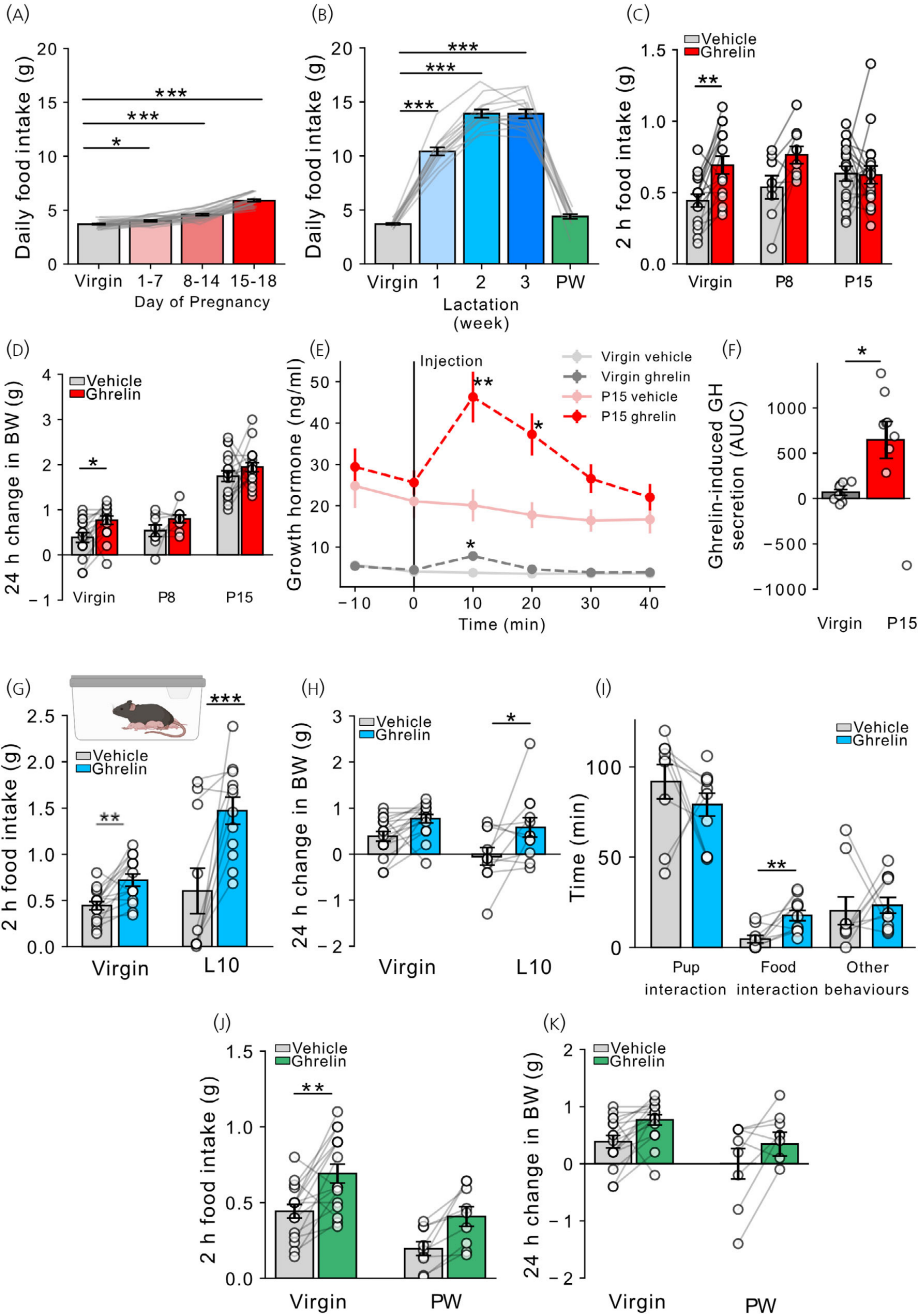
Food intake and body weight changes in response to ghrelin. (A): Mean daily food intake per week in virgin mice and throughout pregnancy (*n* = 24). (B): Mean daily food intake per week in virgin mice, throughout lactation (day 3–20) and post weaning (*n* = 13). (C): Acute food intake following ghrelin or vehicle administration in virgin (*n* = 16), Day 8 pregnant (*n* = 8) and Day 15 pregnant mice (*n* = 18). (D): Change in body weight over 24 h for virgin (*n* = 16), Day 8 pregnant (*n* = 8) and Day 15 pregnant mice (*n* = 18) treated with ghrelin or vehicle. (E): GH levels in virgin and pregnancy mice treated with vehicle or ghrelin (*n* = 9). (F): The GH secretory response (area under the curve, AUC) to ghrelin in the virgin and pregnant mice. (G): Food intake over 2 h in response to vehicle or ghrelin administration in virgin (*n* = 16) and lactating mice (*n* = 10–12). (H): Change in body weight over 24 h in response to vehicle or ghrelin administration in virgin (*n* = 16) and lactating mice (*n* = 10–12). (I): Behavioural analysis of lactating mice over 2 h after vehicle or ghrelin treatment (*n* = 9–11). (J): Food intake over 2 h in response to vehicle or ghrelin administration to virgin (*n* = 16) and mice after their litters are weaned (‘post weaning’) (*n* = 9). (K): Change in body weight over 24 h for virgin (*n* = 16) and post weaning mice (*n* = 9) treated with ghrelin or vehicle.

A series of initial pilot experiments were used to establish treatment parameters in female mice for the study. Virgin female mice responded to exogenous ghrelin treatment with an expected increase in food intake over 2 h (vehicle‐treated 0.44 g ± 0.05 g vs. ghrelin‐treated 0.69 g ± 0.06 g, *p* = 0.003, Figure 2C) and an increase in 24 h body weight gain (vehicle‐treated 0.39 ± 0.11 g vs. ghrelin‐treated 0.77 g ± 0.09 g, *p* = 0.011, Figure 2D). Exogenous ghrelin led to significantly higher GH concentrations 10 min after injection compared to vehicle (vehicle‐treated 3.82 ng/mL ± 0.48 ng/mL vs. ghrelin‐treated 7.87 ng/mL ± 0.85 ng/mL, *p* < 0.001). In response to ghrelin injection, there was a significant increase in the number of AgRP neurons colocalised with c‐Fos compared to vehicle injection (vehicle‐treated 1.185 ± 0.465 vs. ghrelin‐treated 9.88 ± 2.687 neurons per section, *p* = 0.006). These pilot studies established the dose of ghrelin (0.3 mg/kg) and protocols which were then used to assess responses to ghrelin during pregnancy, lactation and post‐weaning period.

**FIGURE 3:**
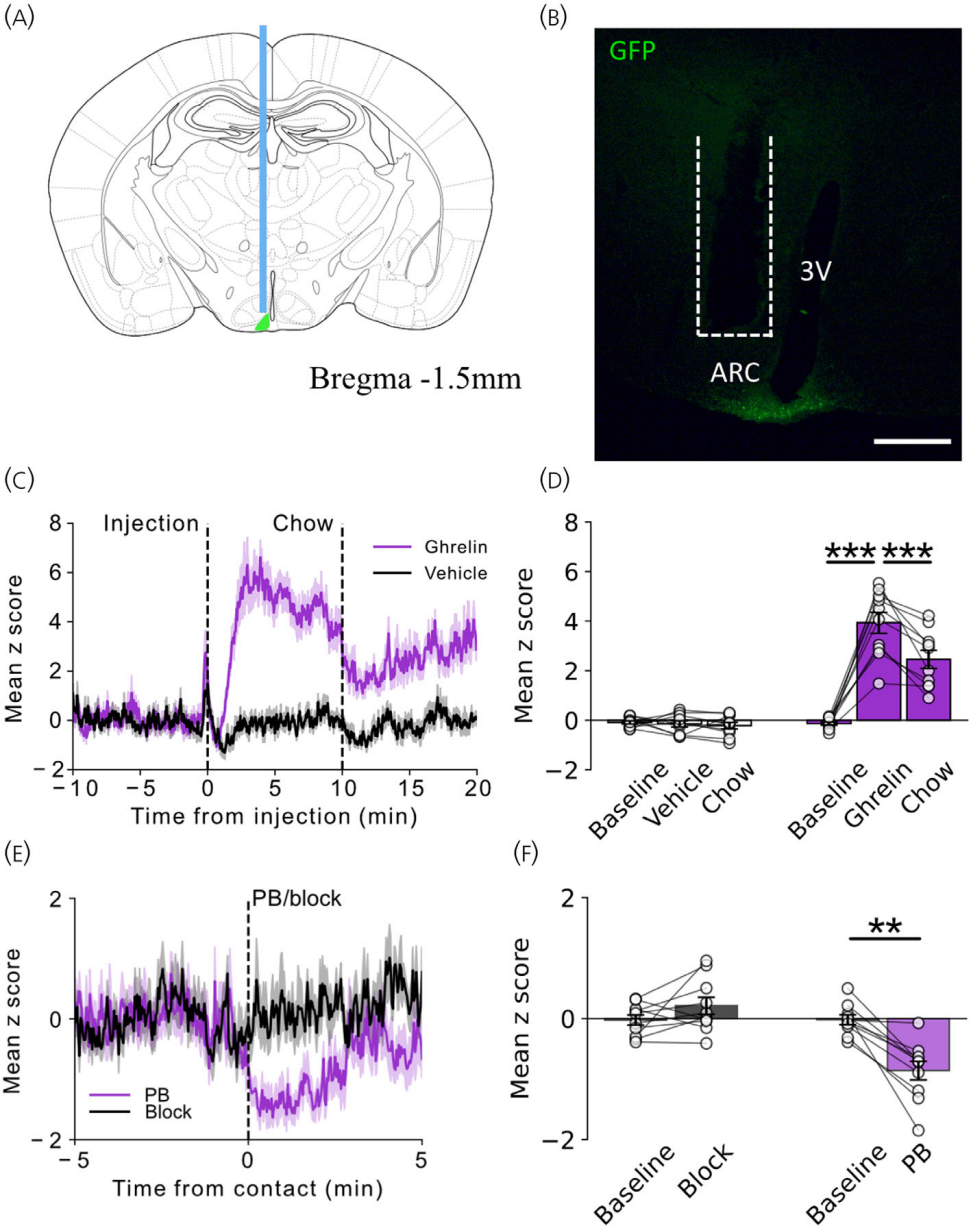
Validation of paradigms for assessment of agouti related peptide (AgRP) neuron population. (A): Schematic of a coronal slice of the brain indicating target location of fibre optic cannula placement for in vivo fibre photometry monitoring of AgRP neurons. (B): Representative image of green fluorescent protein (GFP) immunoreactivity in a coronal brain section through the arcuate nucleus indicating GCaMP6f transduction in AgRP neurons. Scale bar 200 μm, white dotted line represents cannula placement, 3 V third ventricle. (C): Lines indicate mean *z*‐score of AgRP neuron activity (+/− SEM) in response to ghrelin or vehicle administration. Ghrelin increases AgRP neuron activity (purple) while vehicle elicits no response (black). (D): Bars indicate mean *z*‐score +/− SEM along with individual data points (*n* = 10 per timepoint), for the 10 min time bins prior to injection (baseline), following injection of vehicle or ghrelin and then following access to chow. AgRP neuron activity was increased by ghrelin administration then decreased with chow consumption but does not return to baseline within the 10 min. Bars with different letters are significantly different. (E): Lines indicate mean *z*‐score of AgRP neuron activity (+/− SEM) in response to non‐food object or peanut butter chip (PB). AgRP neuron activity decreased in response to PB (purple) but not in response to a plastic cube (black). (F): Bars indicate mean *z*‐score +/− SEM along with individual data points, for the 5 min time bin prior to and following exposure to non‐food object/PB.

**FIGURE 4:**
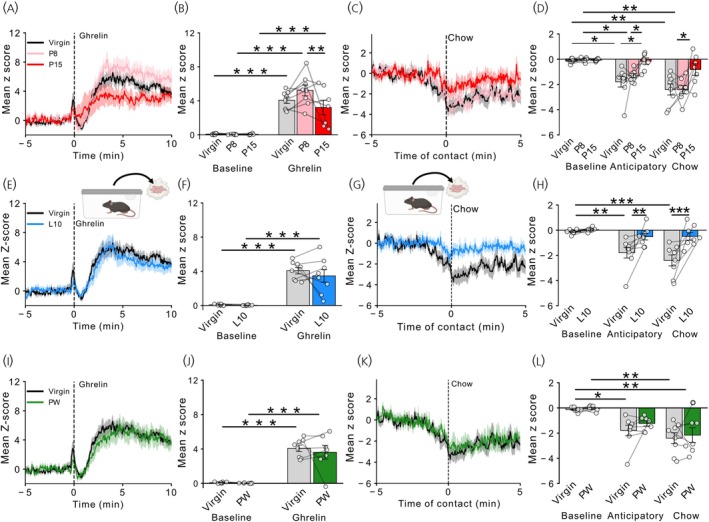
Agouti related peptide (AgRP) neuron activity across reproductive states. AgRP neuronal activity, as measured by in vivo calcium fibre photometry and displayed as *z*‐scores during pregnancy (A–D: *n* = 7–8 per timepoint), lactation (E–H: *n* = 8 per time point) and post weaning state (I–L: *n* = 7–8 per timepoint) compared to the virgin state. Line graphs show continuous *z*‐score throughout the trial, and bar graph show (± SEM) average *z*‐score alongside individual data points. (B): Ghrelin increased AgRP neuron activity during pregnancy similar to virgin mice. (D): Chow decreased AgRP neuron activity during early pregnancy but not during late pregnancy. The chow anticipatory response of AgRP neurons on Day 15 of pregnancy was significantly less than virgin and Day 8 pregnant mice (and the chow response of AgRP neurons on Day 15 pregnancy was significantly less than Day 8 of pregnancy. (E): Ghrelin increased AgRP neuron activity during lactation similar to virgin mice. (H): Chow did not decrease AgRP neuron activity in lactation. The AgRP neuron activity of lactating mice was significantly more than virgin mice in both anticipation of chow and after initiation of chow consumption. (J): Ghrelin increased AgRP neuron activity in the post‐weaning period similar to the virgin mice. (L): Chow decreased AgRP neuron activity in the post weaning state similar to the virgin state.

### 3.2 Food intake and body weight do not increase in response to ghrelin during pregnancy

We observed an increase in daily food intake in both pregnancy and lactation (Figure 2A,B, pregnancy *p* < 0.001, lactation *p* < 0.001) compared with the same animals in the virgin, non‐pregnant state. Within a week following weaning of pups, daily food intake returned to pre‐pregnancy/pre‐lactation levels (Figure 2B, *p* = 0.0552). To determine the acute effects of an exogenous bolus of ghrelin throughout reproductive states, mice were injected (i.p.) with ghrelin or vehicle then 2 h food intake and 24 h change in body weight were measured. While acute injection of ghrelin in virgin female mice significantly increased 2 h food intake compared to vehicle‐treated mice (Figure 2C, *p* = 0.008), on 15 of pregnancy, ghrelin did not increase food intake compared to vehicle‐treated mice at the equivalent stage of pregnancy. Day 8 or pregnancy seemed to be a transitional phase, where most animals did show higher food intake after ghrelin treatment, but unlike the virgin control state, the combined mean was not significantly different between vehicle and ghrelin treatment. Similarly, while ghrelin‐treated virgin mice gained significantly more weight than vehicle‐treated virgin mice in the 24 h after treatment (Figure 2D, *p* = 0.033), at the pregnancy time points both vehicle‐ and ghrelin‐treated groups gained similar amounts of weight. As expected, however, the Day 15 pregnant mice gained more weight over 24 h compared to both virgin and Day 8 pregnant mice (*p* < 0.001) (significant effect of pregnancy, but not of treatment).

Basel GH concentrations were higher on Day 15 of pregnancy compared to the virgin state (*p* < 0.001). Ghrelin increased GH concentration in virgin mice with a peak seen 10 min after injection (Figure 2E, *p* < 0.001). Day 15 pregnant mice also showed ghrelin‐induced GH secretion, with elevated GH concentration at 10 and 20 min timepoints after ghrelin injection (Figure 2E, *p* < 0.001). Despite starting from a much higher baseline, the total GH secretory response to ghrelin compared to vehicle over 40 min was significantly greater in pregnancy compared to the virgin state (Figure 2F, *p* = 0.014).

During lactation, the 2 h food consumption after ghrelin administration increased compared to virgin mice (Figure 2G, *p* = 0.015). Ghrelin‐treated lactating dams also had increased body weight in the 24 h following treatment (Figure 2H, *p* = 0.014). The presence of pups in the lactating groups introduced a confound to interpretation, with many of the vehicle‐treated animals not eating anything at all, but remaining focused on nursing of their pups. This caused significant variability to the food intake data for that group. In a separate cohort, the 2 h period after ghrelin or vehicle administration was video recorded and behavioural analysis demonstrated that ghrelin‐treated lactating mice spent more time interacting with food compared to the vehicle‐treated dams (Figure 2I *p* = 0.002), with a slight reduction in time spent interacting with pups. Of note, in the 2 hour session some vehicle‐treated dams did not leave the pups and nest, but ghrelin administration markedly shifted the preference of dams to seek food before coming back to the nest, consistent with previous literature.^50^

There was no difference in the ghrelin response between the pre‐pregnancy virgin state and mice in the post weaning group, with both groups eating more in the 2 h period after ghrelin treatment compared to vehicle treatment (Figure 2J, interaction *p* = 0.112, effect of treatment *p* < 0.001). Post weaning mice gained less weight over 24 h than had been observed in the virgin state (Figure 2K *p* = 0.005), with or without ghrelin treatment.

### 3.3 Responses of AgRP neuronal activity to various stimuli in different reproductive states

As previously established,^36^, ^39^ in fed virgin female mice, vehicle injection followed by presentation of chow did not affect population activity of the AgRP neurons (Figure 3C,D). In contrast, ghrelin injection led to an increase in AgRP neuron activity that was then reduced after consumption of chow (Figure 3C,D, *p* < 0.001). Contact with a plastic block placed in the cage did not change AgRP neuron activity, while contact with the palatable PB decreased AgRP neuron activity (Figure 3E,F, *p* = 0.002). Using these paradigms, we assessed AgRP population neuronal activity across a complete cycle of pregnancy and lactation, including virgin and post weaning mice (Figure 1).^24^

The mice that underwent surgery for viral delivery of our AAV and fibre optic cannula placement were monitored for oestrous cycle, gestational weight gain and pup weight gains to ensure normal oestrous cycles, pregnancy and lactation. After surgery, mice displayed normal 4–5 day oestrous cycles. In the virgin state, mice had body weights between 21 and 28 g, with a mean of 23.5 ± 0.7 g. Pregnancy body weight gain (Day 1–17) was 10 ± 0.5 g which is in line with body weight data collected in our food intake experiment (Figure 2). Mice gave birth on Day 20 ± 0.2 of pregnancy, and on Day 3 of lactation, average litter size was 6.6 ± 0.2 pups and average litter weight was 9.7 ± 0.3 g. Litter weight gain from Day 3–20 was 40.1 ± 1.2 g. Out of 8 mice that gave birth, none abandoned their pups, and following weaning, dams showed a normal decrease in body weight the first few days post weaning. Overall, the pregnancies and lactation period of the mice used in this study were similar to our previous cohort with no manipulations for photometry (Figure 2) and to our previous work^51^ and indicate healthy pregnancies that were unaffected by our manipulations.

AgRP neuronal activity was assessed in response to ghrelin, chow and PB at two timepoints during pregnancy. On Day 8 (P8) of pregnancy AgRP neuronal activity in response to injection of ghrelin was similar to the virgin state (Figure 4B, *p* = 0.092). Similarly, on Day 15 (P15) of pregnancy, there was still a significant ghrelin‐induced increase in AgRP neuronal activity, although the magnitude of this response was significantly reduced compared to P8 (Figure 4B, *p* = 0.007). Ten minutes after ghrelin injection, mice were presented with chow and AgRP activity showed a different response in Day 15 pregnant mice compared to the virgin and Day 8 mice (Figure 4D, *p* = 0.049). In the virgin and day 8 pregnant mice, presentation of chow caused a decrease in AgRP activity both prior to the consumption of any chow (virgin *p* = 0.015, P8 *p* < 0.001) and following initiation of chow consumption (virgin *p* = 0.004, P8 *p* < 0.001). For the Day 15 pregnant mice, the presentation of chow did not significantly change AgRP activity (*p* = 0.29). The chow anticipatory response of AgRP neurons on Day 15 of pregnancy was significantly less than virgin and Day 8 pregnant mice (virgin vs. P15 *p* = 0.047, P8 vs. P15 *p* = 0.014) and the chow consumption response of AgRP neurons on Day 15 pregnancy was significantly less than Day 8 of pregnancy (*p* = 0.031).

**FIGURE 5:**
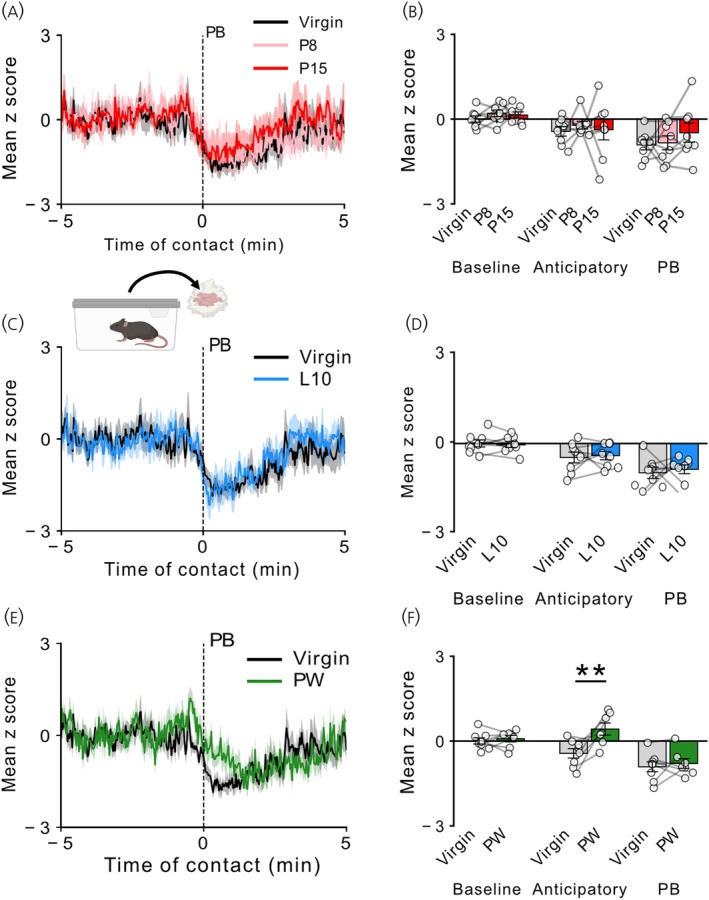
Agouti related peptide (AgRP) neuron activity in response to palatable food across reproductive states. AgRP neuronal activity, as measured by in vivo calcium fibre photometry, in response to peanut butter chip (PB). Line graphs show continuous *z*‐score throughout the trial, and bar graph show (± SEM) average *z*‐score alongside individual data points (*n* = 7–8 per time point). (A, B): Response of AgRP neurons in pregnancy is similar to virgin state. (C, D): Response of AgRP neurons in lactation is similar to virgin state. (E, F): After weaning of their pups, post weaning mice had higher AgRP activity in the presence of PB compared to virgin state.

On day 10 of lactation the response of AgRP neuron activity to ghrelin injection was similar to that in virgin mice (Figure 4F, *p* = 0.43). Unlike in the virgin mice, however, chow did not induce a significant reduction in AgRP neuron activity in lactation (Figure 4H *p* = 0.012). In this paradigm, the activity of AgRP neurons in lactating mice did not change in response to either anticipation (*p* = 0.29) or consumption of chow (*p* = 0.31). Both the anticipatory inhibition of AgRP in response to chow (*p* = 0.003) and the consumption‐induced inhibition of AgRP (*p* < 0.001) were significantly reduced compared with that seen in virgin controls.

After weaning of their pups (post‐weaning), the response of AgRP neurons to ghrelin returned to virgin mice, with ghrelin inducing significant increases in AgRP activity (Figure 4J *p* < 0.001). In post‐weaned mice, the response to ghrelin of AgRP activity was similar to virgin mice (*p* = 0.69). Presentation and consumption of chow led to a rapid reduction in AgRP neuron activity in post‐weaned mice that was indistinguishable to the virgin mice (Figure 4L: anticipatory *p* = 0.22, chow *p* = 0.67).

Virgin, pregnant and lactating mice all showed a similar decrease in AgRP neuron activity in response to presentation and consumption of PB (15 mg) (Figure 5B,D, pregnancy *p* = 0.0003, lactation *p* = 0.0002). In post‐weaned mice, the AgRP neuron activity in response to a PB was different from that in the virgin mice (Figure 5F *p* = 0.044). While both groups had similar activity in the baseline (*p* = 0.73) and the time following initial consumption of PB (*p* = 0.61), the post‐weaned mice had significantly greater AgRP activity in the anticipatory phase compared to the virgin mice (*p* = 0.0009).

**FIGURE 6:**
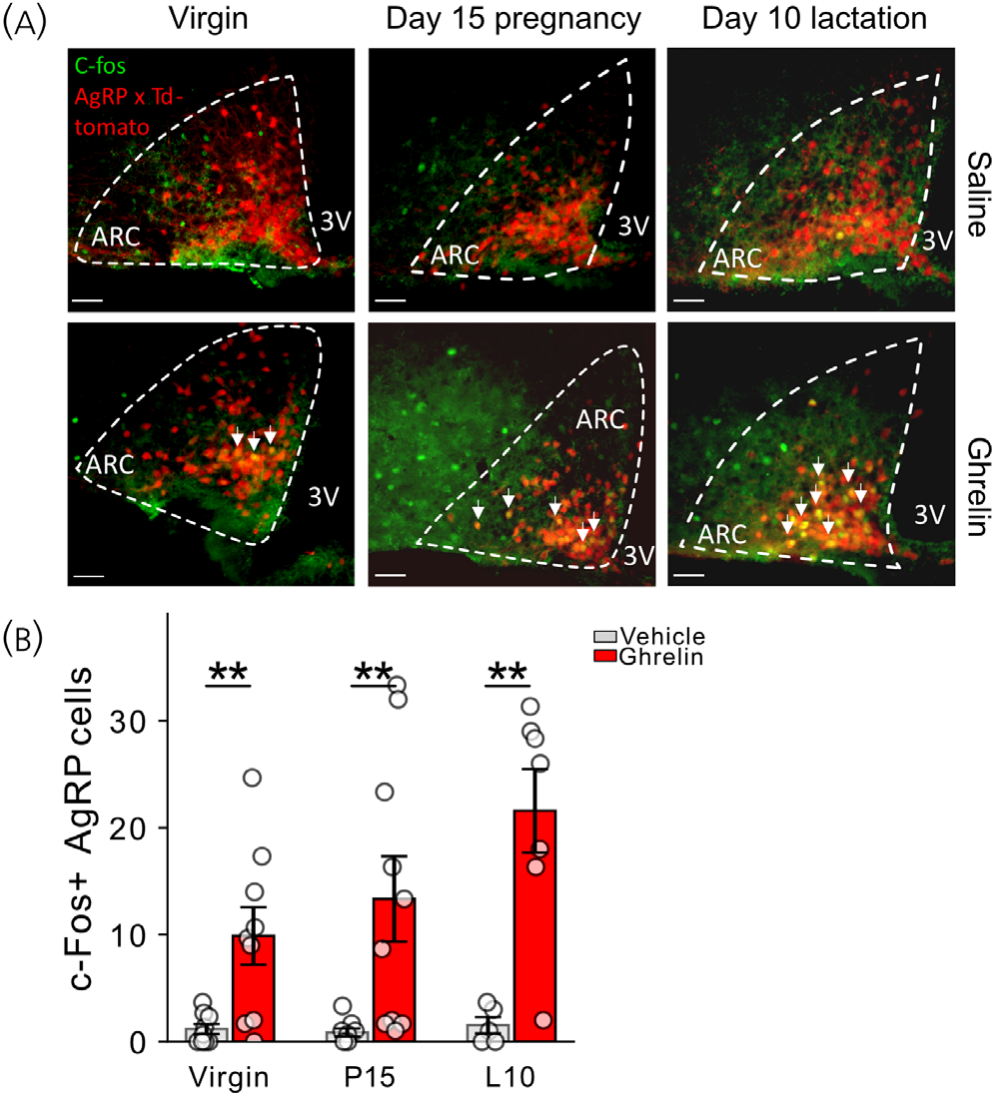
Ghrelin‐induced activation of agouti related peptide (AgRP) neurons. (A): Representative images of endogenous td‐Tomato indicative of AgRP neurons (red) and c‐Fos immunostaining (green) in the arcuate nucleus after vehicle or ghrelin administration across reproductive states. White arrows indicate co‐localisation. White dashed line indicated nucleus boundary. Scale bar 200 μm, 3 V third ventricle. (B): c‐Fos expression co‐localised with AgRP neurons in response to vehicle or ghrelin treatment at virgin (*n* = 9), pregnancy Day 15 (*n* = 9–10) and lactation Day 10 (*n* = 5–7) timepoints.

### 3.4 Ghrelin‐induced an increase in c‐Fos in AgRP neurons in pregnancy and lactation

The number of ghrelin‐responsive AgRP neurons in pregnancy and lactation was assessed using ghrelin‐induced c‐Fos immunofluorescence in transgenic mice that express td‐Tomato in AgRP neurons. An increase in ghrelin‐induced colocalisation of c‐Fos with AgRP neurons was detected in both pregnancy and lactation compared to vehicle (Figure 6B, pregnancy Day 15 *p* = 0.004, lactation Day 10 *p* < 0.001).

## 4 DISCUSSION

In this study, we examined the role of ghrelin in maternal food intake. Surprisingly, ghrelin administration did not acutely increase food intake during pregnancy, despite a ghrelin‐induced increase in AgRP neuronal activity at Day 8 and 15 of pregnancy and increased overall food intake. These results suggest a form of ghrelin resistance develops during pregnancy, at least downstream of AgRP neurons. The observed ghrelin resistance was specific to the food intake response, as ghrelin‐induced GH secretion was maintained or even enhanced during pregnancy. The total amount of food eaten in response to ghrelin was unlikely to be limited by the fact that the pregnant animals were already eating more, because the experiment was done in the light period when they don't eat much, and we showed that the absolute amount eaten was much higher in lactation, yet they still showed a marked response to ghrelin at the time. By Day 10 of lactation, ghrelin‐induced food intake was significantly elevated compared to virgin, demonstrating increased sensitivity to acute ghrelin administration. This ghrelin sensitivity likely contributes to the increased food intake in lactation. After weaning, ghrelin‐induced food intake and AgRP neuronal activity response returned to normal levels, indicating that pregnancy is a novel physiological state of transient and targeted ghrelin resistance. While ghrelin increased AgRP neuronal activity equally in virgin and lactating mice, the acute fall in activity in response to consuming chow was significantly attenuated in lactating mice. Thus, during lactation AgRP neurons may be less sensitive to post‐ingestive satiety signals leading to increased meal duration and increased food intake.^52^ Collectively, our results show the function of ghrelin undergoes adaptive changes through the course of pregnancy and lactation in female mice, with ghrelin resistance occurring during pregnancy and increased sensitivity to ghrelin during lactation.

Pregnancy is a state of positive energy balance, incorporating not only the immediate demands of energy for the developing foetus and adaptations of maternal tissue, but also increased fat deposition, presumably as an anticipatory source of energy in preparation for future metabolic demands during late pregnancy and lactation. The increase in energy demands during pregnancy is associated with elevated food intake.^51^ During pregnancy, many of the satiety hormones no longer induce an acute reduction in food intake, suggesting that lowered sensitivity to satiety hormones may facilitate elevated maternal food intake.^1^ Since ghrelin stimulates food consumption,^9^ we hypothesised that increased ghrelin sensitivity would contribute to increased food intake during pregnancy. However, the current study demonstrates that ghrelin did not acutely increase food intake during pregnancy, indicating that ghrelin resistance to food intake develops during pregnancy, similar to that seen with diet‐induced obesity.^53^ Interestingly, plasma leptin and body weight gain contribute to ghrelin resistance during diet‐induced obesity^54^ and both plasma leptin and body weight are increased during pregnancy. Moreover, the loss of fat mass associated with diet restriction after high‐fat feeding correlated with increased ghrelin sensitivity.^55^ Taken together, the increase in plasma leptin and fat mass during pregnancy is likely to underpin ghrelin resistance during late pregnancy.

On the other hand, lactation places a significant energy demand on the mother causing a state of negative energy balance, in which a dramatically increased energy intake is required. Our results demonstrate that lactating mice are more sensitive to the food intake‐stimulating effects of ghrelin, as previously described.^52^ The re‐sensitisation of lactating mice to ghrelin is similar to diet‐restriction in obese mice, as a decrease in fat mass and plasma leptin improves ghrelin responsiveness.^55^, ^56^, ^57^, ^58^ Thus, the effectiveness of ghrelin during pregnancy or lactation is consistent with metabolic state, characterised by changes in fat mass and plasma leptin controlling the sensitivity of the ghrelin response.^59^

These data demonstrate that lactation and pregnancy both require elevated food intake, but during pregnancy food intake drives weight gain and adiposity (positive energy balance) whereas during lactation, food intake prevents a loss of weight and fat mass (negative energy balance). Indeed, the actions of ghrelin are attenuated during positive energy balance and potentiated during negative energy balance, consistent with the role of ghrelin as a hormonal defence against starvation.^59^, ^60^, ^61^

AgRP neurons stimulate food intake and are a key target for the actions of ghrelin.^23^, ^36^ High‐fat diet (HFD)‐induced obese male mice are insensitive to ghrelin, as determined by attenuated ghrelin‐induced c‐Fos^53^ and attenuated ghrelin‐induced stimulation of AgRP neuron population activity monitored in vivo via GCaMP photometry.^37^ However, during pregnancy or lactation, ghrelin‐induced AgRP neuronal activity was largely unaffected, as assessed either by c‐Fos or AgRP neuronal population activity. There was a small reduction in Ghrelin‐induced AgRP activity in late pregnancy compared with mid pregnancy, but the magnitude of response in late pregnancy was comparable to that in virgin mice. It seems likely that this change in response during late pregnancy is due to rising levels of oestradiol, with an increase from mid pregnancy until birth,^62^, ^63^ as oestradiol attenuates ghrelin‐induced food intake.^64^ Overall, however, neither the attenuated or heightened sensitivity to the feeding response to ghrelin during pregnancy and lactation respectively, could be ascribed to major changes in how ghrelin activates AgRP neurons during these physiological states. This is different from the mechanism seen in diet‐induced obesity, as AgRP neurons become unresponsive to ghrelin at this time and weight loss restores this AgRP sensitivity to ghrelin.^37^, ^53^ Therefore, we propose that pathways downstream of AgRP neurons mediate the attenuated and heightened sensitivity to ghrelin‐induced food intake during pregnancy and lactation respectively. Indeed, there is evidence of prolonged feeding effects in the absence of AgRP cell body stimulation that is mediated by NPY release in nerve terminal regions^30^ and i.c.v. NPY injection increases food intake in diet‐induced obesity.^53^ In addition, synaptic release of neurotransmitters from AgRP neurons is important for normal control of food intake.^43^ AgRP activity can also alter post synaptic activity in downstream nerve terminal regions,^65^ which in some regions is absent in NPY deficient mice.^66^ Collectively, we suggest the changes in ghrelin sensitivity during pregnancy and lactation are driven by pre‐synaptic changes in nerve terminal regions of AgRP neurons, rather than changes in activity at cell bodies. It is also possible that the first stages of ghrelin resistance occur within the nerve terminal, which then propagates back to affect cell body activity over time. In support for this idea comes from the time course for the development of ghrelin resistance in obesity; the first signs of ghrelin resistance occurred after 3 weeks^55^ which is approximately the length of pregnancy in mice. Thus, the 3‐week pregnancy maybe enough to suppress ghrelin‐induced food intake, but not long enough to also cause suppression of AgRP activity by ghrelin, which has been observed after 6 weeks of high fat diet feeding.^37^

Ghrelin has many other functions beyond food intake, including increasing gastric motility and emptying, increasing cardiac output and vasodilation, decreasing thermogenesis in brown adipose tissue and increasing GH secretion.^67^ In this study, ghrelin‐induced GH release remained intact during pregnancy, indicating that pregnancy‐induced insensitivity to ghrelin may be selective to its effects on food consumption. Another possibility is ghrelin resistance had not progressed long enough to affect other aspects of ghrelin function as we previously observed reduced GH secretion associated with ghrelin resistance in diet‐induced obese mice that had been on a HFD for 12 weeks.^53^ Interestingly, basal GH levels were increased in pregnancy, consistent with previous literature,^68^ but the ghrelin‐induced release of GH was greatly amplified compared to the virgin state, possibly due to increased expression of the GHSR.^69^, ^70^ However, it seems unlikely that this elevated response of GH to ghrelin contributes to the significantly higher levels of GH during pregnancy,^68^, ^71^, ^72^ given that GH concentrations during pregnancy and lactation are not affected in a mouse model lacking acylated ghrelin.^72^

In both late pregnancy and lactation, there was an attenuated response of AgRP neurons to the presentation and consumption of chow. In males and virgin females, the presentation of chow rapidly decreases AgRP neuronal activity.^24^, ^39^ Initially sensory signals such as sight and smell of food transiently inhibit AgRP neuron activity, then post‐ingestive gastrointestinal signals following consumption maintain inhibition of AgRP neurons.^42^, ^73^ Both the anticipatory response to chow presentation and the consummatory response to chow ingestion were attenuated in late pregnancy and lactation suggesting alterations in the sensory processing of information reaching AgRP neurons may affect the anticipatory response. For example, the olfaction system undergoes adaptations during pregnancy and lactation relating to the onset of parental behaviour^74^, ^75^ and AgRP neurons^76^, ^77^ can influence olfactory sensitivity. During consumption, the greater need for energy intake in pregnancy and lactation may underlie the attenuated reduction in AgRP neuron activity. Both late pregnant and lactating mice increase their overall food intake by having larger/longer meals or bouts of eating,^51^, ^52^ suggesting they are less sensitive to post‐ingestive neural or peptide feedback signals initiating satiation. Indeed, the suppression of AgRP activity following food intake can be replicated with gut peptides signalling satiety in a concentration dependent manner.^42^, ^73^ For example, cholecystokinin (CCK) at 30 μg/kg produces a much greater fall in AgRP activity than at 3 μg/kg and during pregnancy the satiety‐inducing effects of CCK are significantly reduced.^78^ Thus, the attenuated suppression of AgRP activity to food during pregnancy and lactation may reflect reduced sensitivity to feedback signals of satiety, which ultimately ensure larger/longer meals or bouts of eating^51^, ^52^ and therefore increased food intake. However, previous studies show that chow presentation and consumption lead to greater inhibition of AgRP neurons after 10 h of fasting in lactating compared to non‐lactating mice.^51^, ^52^ In our study, AgRP neuron activity was assessed following ghrelin‐induced food intake, thus the differences between these two studies may reflect the hunger state (long‐term fasted with associated stress vs. acute hunger induced by exogenous hormone). Nevertheless, these two different effects observed in lactation reflect a rapid ability of AgRP neurons to adapt their responses to alterations in physiological state during lactation.

Overall, this study extends previous work showing pregnancy‐induced adaptive changes in body weight homeostasis, with pregnancy being a state of resistance to not only satiety effects of the major peripheral signals such as leptin, insulin and CCK^1^, ^78^, ^79^ but also the appetite stimulating effect of the orexigenic hormone ghrelin. This resistance is specific to pregnancy, as the food intake response to ghrelin is regained postpartum. The GH‐releasing effect of ghrelin and the acute responses of AgRP neurons to ghrelin were unaffected during pregnancy, indicating that the selective loss of food intake response to ghrelin is downstream of activation of AgRP neurons. The combined loss of sensitivity to both orexigenic and satiety signals during pregnancy essentially means that food intake is unregulated by the normal homeostatic controls during pregnancy, potentially a hormone‐driven adaptive change to allow development of a positive energy balance state that is specific to pregnancy.

## AUTHOR CONTRIBUTIONS

**C. L. Murrell:** Conceptualization; investigation; writing – original draft; methodology; formal analysis; data curation. **M. R. Perkinson:** Methodology; Formal analysis; data curation; writing ‐ review and editing. **D. R. Grattan:** Conceptualization; writing – review and editing; supervision. **Z. B. Andrews:** Conceptualization; writing – review and editing; supervision. **S. R. Ladyman:** Conceptualization; investigation; funding acquisition; writing – original draft; writing – review and editing; supervision; resources; project administration; formal analysis.

## FUNDING INFORMATION

This work was supported by the Health Research Council of New Zealand (Grant number 22/248) and the Malcolm Templeton Fund for Neurological Research (University of Otago).

## CONFLICT OF INTEREST STATEMENT

The authors declare no conflict of interest.

## Supporting information


**Table S1:** Table of statistical tests.

## Data Availability

The data that support the findings of this study are available from the corresponding author upon reasonable request.
